# Relationship of Serum Prestin Levels to the Severity of Sensorineural Hearing Loss

**DOI:** 10.7759/cureus.53763

**Published:** 2024-02-07

**Authors:** Ali Rasheed H Al Samarrai, Bakr M Mahdi, Kourosh Parham

**Affiliations:** 1 Department of Biology, University of Samarra, College of Education, Samarra, IRQ; 2 Department of Otolaryngology, Samarra General Hospital, Samarra, IRQ; 3 Department of Otolaryngology-Head and Neck Surgery, University of Connecticut Health, Farmington, USA

**Keywords:** enzyme-linked immunosorbent assay (elisa), cochlea, biomarker, outer hair cells, prestin, sensorineural hearing loss

## Abstract

Objective: Prestin is an outer hair cell (OHC) protein responsible for increasing cochlear sensitivity and has been proposed as a biomarker. We aimed to evaluate whether the serum prestin level is related to the severity of chronic sensorineural hearing loss (SNHL).

Methods: Ninety subjects were recruited from the patient base at Samarra public hospitals and clinics in Iraq from January to October of 2022. They were divided into three groups equally: a group of healthy people without hearing loss (G0), a group with moderate SNHL (G1), and a group with severe SNHL (G2). The subjects ranged from 20 to 80 years of age and included 51 males and 39 females. Blood samples were collected, then serum was separated, and enzyme-linked immunosorbent assays were performed to quantify the levels of prestin.

Results: Hearing thresholds were sequentially statistically higher across the three groups. While prestin levels were significantly higher in G1 and G2 than that in G0, there were no differences between the G1 and G2 levels. Serum prestin levels were positively correlated with hearing thresholds in G1, but not G2.

Conclusion: Our results suggest that in the clinical setting, prestin is sensitive to chronic mild to moderate SNHL (i.e., up to 40-60 dB), not more severe loss. This range is consistent with the added sensitivity provided by OHCs in the cochlea and provides support for prestin as a biomarker of OHC-mediated SNHL.

## Introduction

Inner ear disorders represent a challenge due to the limited diagnostic and treatment options being available. One of the limitations in otology has been the absence of biomarkers that can guide management. Prestin was first proposed as a possible biomarker based on a hypothesis that key inner-ear-specific proteins that bestow its highly specialized function may inform on the health of the cochlea and more specifically outer hair cells (OHCs) [1]. Prestin subserves OHC functions of improving cochlear tuning and amplification, two processes that are critical to healthy hearing. There have been promising experimental results in noise- and ototoxin-induced models [2-7] that have supported a promising role for prestin as a biomarker. While human studies have also yielded support for the concept [8-14], additional results are needed to help translate prestin as a biomarker from the research setting to the clinical setting. This is particularly important since prestin is also expressed by cardiac muscles, where it serves to amplify cardiac function [15]. More studies are needed in humans to establish the parameters that influence serum prestin. We have shown its reliability and stability over time [16], negative correlation with daily noise exposure [17] in normal hearing young adults, and its decline with age per se [18]. Recognizing that previous experimental research demonstrated that OHCs provide 40-60 dB of sensitivity [19], here, we examine serum prestin levels in chronic sensorineural hearing loss (SNHL) in relation to the severity of hearing loss, controlling for age.

## Materials and methods

Participants and exclusionary criteria

All experimental procedures were approved by the Institutional Review Board at Samarra University and Samarra General Hospital (#3-7-96). Ninety subjects (33 males and 67 females), between 20 and 80 years of age, were recruited from the patient base at Samarra public hospitals and clinics from January to October of 2022. Exclusion criteria were history of or presence of otologic diseases, such as chronic suppurative otitis media and its complications; history of head injuries; asymmetric (>10 dB difference between two ears at more than one frequency) or conductive (>5 dB at more than two consecutive frequencies or more than three non-consecutive frequencies) hearing loss; past ear surgeries; exposure to ototoxic drugs; radiation exposure in the head and neck region, autoimmune diseases; tympanic membrane perforation; and diabetes and hyperlipidemia. The study population was divided into three groups: G0 (without hearing loss), G1 (with mild to moderate SNHL (20-55 dB HL)), and G2 (with moderately severe-to-profound SNHL (>55 dB HL)). Each group consisted of 30 subjects.

Audiometric testing

Tympanometry test was performed at the acoustic frequency of 226 Hz. Only subjects with type A tympanograms were included. Hearing thresholds were measured using a conventional audiogram. Otoscopy was performed to examine the auditory canal and eardrum to ensure the absence of earwax, tympanic membrane perforation, infection, or congenital abnormalities. The subject was placed in a soundproof room. Headphones were placed to measure air conduction (AC) thresholds. Tones of varying intensity and frequency were presented to each ear separately, and the level was increased by 5 dB or more. Test frequencies were 0.25, 0.5, 1, 2, 4, and 8 KHz. Bone conduction (BC) measurement was carried out by placing the vibrating device behind the pinna. The same test frequencies as AC were presented.

Blood draw procedures

Blood samples were collected by drawing 5 ml of venous blood, and then the samples were stored at room temperature until coagulation was complete. The supernatant was centrifuged for 10 minutes at 3000 rpm/min, and then the serum was withdrawn and frozen at -80 °C. The samples were subjected to a serum assay using enzyme-linked immunosorbent assay (ELISA) to quantify the level of prestin (Cat. No. E4170hu, BT LAB, Shanghai, China; range: 10-3,000 pg/mL, sensitivity: 4.87 pg/mL) as described in the manufacturer’s instruction manual.

Statistical analyses

In this study, the AC and BC thresholds were averaged across both ears. Statistical analyses were carried out using the statistical software IBM SPSS Statistics for Windows, version 26 (released 2019; IBM Corp., Armonk, New York, United States). Log transformation was performed to normalize distributions of prestin. The results were analyzed for statistical significance using analysis of variance (ANOVA), and follow-up testing was carried out using Tukey's HSD (honestly significant difference). Pearson’s correlation coefficient (r) was used to evaluate the relationship between various variables. Statistical significance was set at p < 0.05 (one-tailed when the direction of difference or relationship was known). Finally, a regression analysis was carried out with prestin as the dependent variable with age and pure tone audiometry (PTA) as predictors.

## Results

**Figure 1:**
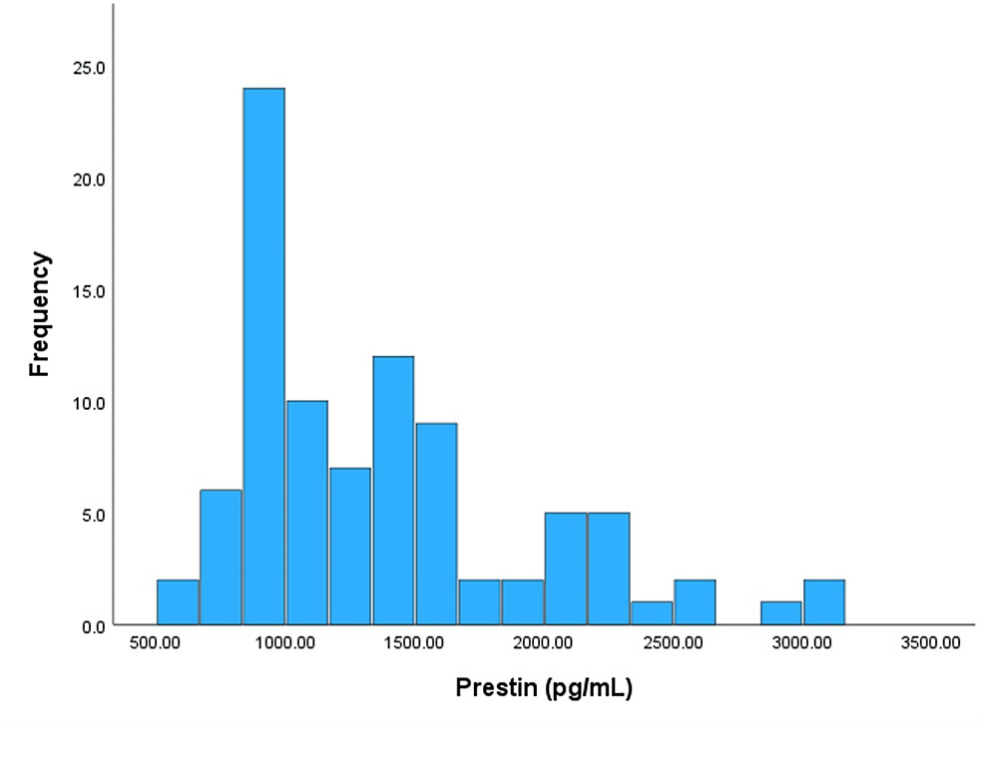
Distribution of prestin levels across the entire study population.

The subjects’ PTAs ranged from 7.9 to 113.3 dB HL. Prestin levels ranged from 533.1 to 3110.6 pg/mL (Figure 1). The prestin distribution was skewed. To normalize the variable, as we had done in our previous human studies, log transformation was carried out.

**Figure 2:**
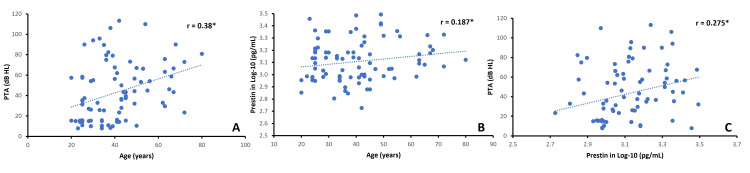
Relationships of PTA and age (panel A), prestin level and age (panel B), and prestin level and PTA (panel C). Each panel includes a dotted trend line. r is the Pearson correlation coefficient; *one-tailed p < 0.05.

Figure 2 shows the relationship of PTA to age (panel A) and those of prestin levels to age (panel B) and PTA (panel C). There were weak to moderate correlations between age and PTA, age and prestin level, and prestin level and PTA, with the Pearson correlation coefficient ranging from 0.19 to 0.38. These correlations were statistically significant (one-tailed p < 0.05).

In a regression analysis, p = 0.051 with R^2^ = 0.066 (6% of variance in the prestin level is accounted for by PTA and age). Between PTA and age, PTA was the more important predictor of prestin, such that as PTA increases by 1 dB HL, prestin increases by 4.3 pg/mL (p = 0.052).

Table 1 shows the study participants according to group assignment, gender, sample size, age, and mean PTA. Males outnumbered females in G0 and G1. Males and females did not have a statistically significant difference in age or PTA within any of the three groups. Males and females were pooled together in each group for the remaining analyses. The average age was 31.67, 43.13, and 45.13 years in G0, G1, and G2, respectively. G0 was significantly younger, followed by G1 and G2 in age (p < 0.05), but there was no significant difference between G1 and G2.

**Table 1:** Mean results of the audiometry of both ears in the three groups. SEM = standard error of the mean, PTA = pure tone audiometry

Group	Gender	N	Mean age (years)	SEM	Mean PTA (dB HL)	SEM
G0		30	31.67	1.04	13.24	0.52
	Male	18	29.88	1.51	13.06	0.77
	Female	12	34.16	2.78	13.55	0.62
G1		30	43.13	3.74	37. 85	1.87
	Male	13	43.70	3.89	41.51	2.90
	Female	17	43.47	3.54	35.04	2.28
G2		30	45.13	2.91	76.68	3.11
	Male	20	43.05	2.78	76.89	3.93
	Female	10	49.30	6.32	76.25	5.29

The pooled PTAs for G0, G1, and G2 were 13.2 ± 0.5, 37.8 ± 1.88, and 76.68 ± 3.1 dB HL (mean ± SEM), respectively. The main effect of the groups was significant in a one-way ANOVA (F (2,87) = 228.5, p < 0.001). Follow-up testing showed that the PTAs of all three groups (p < 0.001) were significantly different, with G2, having the highest PTAs of all three groups. 

**Figure 3:**
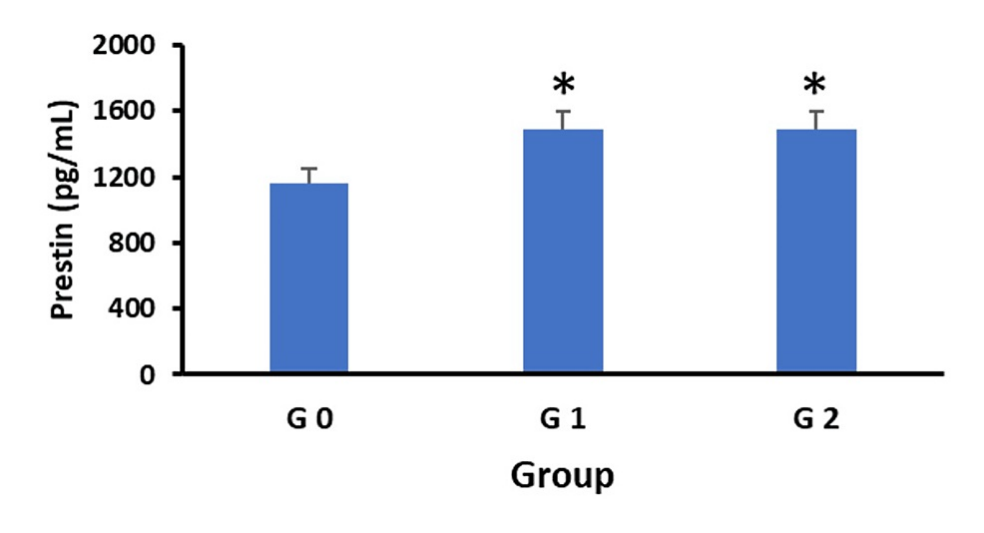
Prestin concentrations in the three groups: G0, hearing healthy; G1, mild to moderate SNHL and G2, moderately severe-to-profound SNHL. Error bars represent SEMs. *Statistically significant difference relative to G0 (one-tailed p < 0.015).

Figure 3 shows the serum prestin levels of each of the study groups. The prestin levels in the hearing-healthy subjects, G0, averaged 1162.82 pg/mL. Prestin levels increased between G0 and G1, with no further rise when G1 was compared to G2. The main effect of the groups was significant in a one-way ANOVA (F(2,87)= 3.52, p = 0.034). Follow-up Tukey HSD testing showed that the prestin levels were significantly higher in G1 and G2 than G0 (one-tailed p < 0.05), but there was no difference between G1 and G2. The percentage increase in the prestin level in the two hearing loss groups compared to healthy subjects was about 28%.

**Figure 4:**
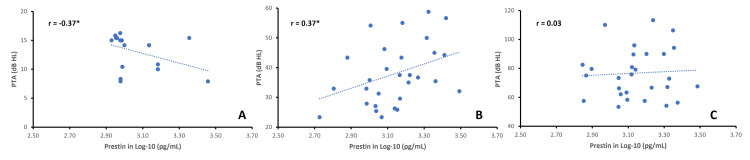
Scatter plots showing the relationship between the prestin levels and PTA in the control (A), G1 (B), and G2 (C). Each panel includes a dotted trendline. r is the Pearson correlation coefficient, *one-tailed p < 0.05.

Figure 4 shows the relationship between prestin levels and PTA across groups. There was a moderate correlation between the two measures in G0 and G1, but not in G3. The direction of the correlation was negative in G0, but this is likely the result of a handful of subjects with prestin levels greater than 3.1 as the distribution was heavily skewed towards lower end of the prestin range. Therefore, the significance of this relationship is not clear. The relationship however was positive and more robust in G1 (r = 0.37) (one-tailed p < 0.023). No correlation between PTA and prestin level was present in G2.

## Discussion

Nearly 10 years ago, we proposed the concept of inner ear proteins as biomarkers of inner ear health [1,20]. There are several proteins with key structural and functional roles, which may serve as biomarkers [21]. Our group has been primarily focused on prestin as a biomarker for SNHL. While experimental models provided a useful proof of concept, presentations of inner ear disorders are more complex in humans because of difficult-to-control modifying factors. In human subjects, we previously explored prestin levels toward differentiating patients presenting with asymmetric SNHL due to acoustic neuromas vs. idiopathic sudden SNHL [22], as well as Meniere’s disease vs. vestibular migraines [23], as have other independent groups [9,14].

We also previously demonstrated that in young, normal-hearing subjects, prestin levels were stable over time [16], positively correlated with otoacoustic emission levels [16], and negatively correlated with daily noise exposure [17]. Young adults at risk of noise-induced hearing loss were distinguished from their not-at-risk peers by their prestin levels when OAE levels were the same [17]. We have also found age-related effects in prestin levels in normal hearing older adults [18].

Here, we systematically investigated prestin levels in subjects with varying severity of SNHL. The main findings of this study are as follows: 1) serum prestin levels increase with age in the presence of SNHL; 2) serum prestin levels increase in the presence of mild-to-moderate SNHL, but do not increase further with increased severity of SNHL; and 3) serum prestin levels are positively correlated with PTA for those with mild-to-moderate SNHL.

The finding of age-related increase in prestin level appears to differ from our recent report of age-related decrease [18]. There is an important difference in the study populations of the two studies. Here, most of the older subjects had moderately severe to profound SNHL. In our previous study, we intentionally recruited subjectively-normal-hearing subjects [18]. Thus, the difference between the results of the two studies may reflect the interaction between aging and SNHL. In another study, where we did not restrict subject recruitment for hearing, we observed an increase in blood otolin-1 (another potential biomarker for inner ear health) [24] and prestin [unpublished] levels, similar to that seen here, supporting a view that blood levels of these biomarkers behave differently under distinct scenarios of aging vs. presbycusis.

Another group has also reported that age and prestin were negatively correlated [25] as we had previously reported [18]. Others have found increased levels of both prestin and otolin-1 levels in patients with noise-induced SNHL [8] and decreased levels of both biomarkers in patients with lead toxicity [10]. In cisplatin ototoxicity, one study reported a dose-dependent rise in prestin levels during within one to three weeks of treatment in cancer patients [11], while another reported decreased levels within 90 days after cisplatin exposure [13]. We believe that diverging results arise from differences in subject populations, temporal considerations in serum sampling, and challenges in controlling for all disease-modifying factors in clinical investigations.

Another important variable that may influence the findings of otologic biomarker investigations is acute vs. chronic presentation. All the experimental model studies that have provided proof of concept for otologic biomarkers have been carried out in the acute setting using noise or ototoxins. These studies provide important information on the quantitative and temporal patterns of changes that occur in the biomarker levels and are needed toward the interpretation of the results in the clinical setting. To better understand and interpret the changes described in humans, under chronic and/or progressive conditions, experimental studies are needed beyond a few days to months after damage has been induced to characterize biomarker changes that follow.

The correlations between hearing levels and biomarkers reported here are replications of our findings of correlations with audiometric measures, including OAEs [16] and hearing thresholds [18]. While this is an important hallmark of an inner ear biomarker, it should be emphasized that these correlations are at best moderate. An explanation offered by the present results is that the moderate correlation is evident in mild-to-moderate SNHL only and decreases in those with more severe SNHL (Figure 4). This was also evident in our finding that serum prestin levels increased in the presence of mild-to-moderate SNHL but did not increase further with increasing severity of SNHL (Figure 3). 

Our study is similar in design to that of Asli et al. [12]. Our findings diverge in that Asli et al. concluded that the prestin level was significantly associated with the severity of hearing loss, whereas we found that prestin levels were not significantly associated with the severity of hearing loss beyond the moderate SNHL range. Asli et al.’s mild and moderate SNHL groups had nearly three times the number of subjects in their severe SNHL, with the latter group having less than 30 subjects. We suspect that unequal sample size may have influenced their conclusions.

Serum prestin being sensitive to SNHL is also the conclusion of a recent study by Emre et al., which included young- and old-control groups compared to young- and old-hearing impaired groups, showing that hearing loss resulted in significant changes in serum prestin levels, but age did not [25]. As noted above, our study provides additional insights in that it shows the sensitivity of prestin to SNHL decreases beyond moderately severe loss (i.e., up to 60 dB HL). This is consistent with an experimental study that demonstrated OHC electromotility in vitro is lost and a 40-60 dB loss of cochlear sensitivity occurs in vivo after the deletion of the prestin gene [19]. Since higher hearing thresholds greater than 40-60 dB are likely to involve mechanisms beyond prestin/OHCs (e.g., inner hair cells), the lack of sensitivity to higher levels of loss provides internal consistency.

When we originally proposed prestin [1] as a biomarker of inner ear health, we postulated that changes in their blood levels could occur either as a result of direct damage (e.g., the release of prestin from dying OHCs) or altered expression of cochlear proteins induced by deleterious agents. Our findings, in the setting of a chronic condition, seem to support the latter hypothesis. Specifically, age- and SNHL-related increases in the biomarker levels may reflect a compensatory upregulation of the expression of inner ear proteins in the surviving hair cells. While experimental biomarker data on aging and presbycusis are not currently available, there is supportive evidence elsewhere. In surviving OHCs of noise-induced hearing loss models, the prestin gene [26] and mRNA expression [27] increased. In an ototoxicity model, chronic treatment with aspirin reversibly increased the expression of prestin in OHCs [28]. Similar changes have also been reported in OHCs after radiation exposure [29].

A limitation of this study is that the group without hearing loss was about one decade younger than either of the hearing loss groups, which were not significantly different from one another in age. This may be viewed as a confounding variable raising concern that prestin changes between hearing-healthy and hearing-impaired groups may be due to age. Our previous study on age-related changes in the blood levels of prestin offers some insights [18]. In that study, an age-related decrease was found in prestin levels above 50 years of age. By contrast, here, we report an increase in prestin levels, the difference between the two studies being the severity of SNHL. Furthermore, we carried out a regression analysis and found that the influence of age was minor compared to the hearing threshold with a 1 dB HL increase, resulting in a 4.3 pg/mL increase in the prestin level, primarily in the mild to moderate SNHL range (Figure 4B).

Another limitation of this study is that we did not take into account the cardiovascular health of our subjects. Hearing loss has been associated with cardiovascular disease and its risk factors [30]. Thus, cardiovascular diseases may influence prestin levels and should be controlled for in future clinical investigations. As we previously noted, prestin is also expressed by cardiac muscles [15]; thus, serum prestin may not be exclusively of otologic origin. At present, it is not possible to differentiate the origins of serum prestin, but such efforts could increase the specificity of prestin as an otologic biomarker. Finally, further investigations could explore the expression of prestin in both the inner ear and heart as one of the possible mechanisms underlying the association between SNHL and heart diseases.

## Conclusions

Our results further validate the role of prestin as a biomarker for SNHL in the chronic setting. We also highlight that the greatest value of this novel biomarker is within the range of hearing loss, which may be primarily attributed to OHC disruption and loss. In more severe hearing loss, the likely involvement of other cellular mediators (e.g., inner hair cells, spiral ganglion cells, and stria vascularis) diminishes the role of prestin as a biomarker.
